# First-Line Treatment in EGFR Mutant Non-Small Cell Lung Cancer: Is There a Best Option?

**DOI:** 10.3389/fonc.2018.00094

**Published:** 2018-04-10

**Authors:** Ajaz Bulbul, Hatim Husain

**Affiliations:** ^1^Department of Hematology/Oncology, Texas Tech University Health Sciences Center School of Medicine, Lubbock, TX, United States; ^2^Division of Hematology Oncology, Kymera Independent Physicians, Roswell, Carlsbad, Hobbs, NM, United States; ^3^University of California San Diego, Moores Cancer Center, La Jolla, CA, United States

**Keywords:** lung cancer, epidermal growth factor receptor, targeted therapy, osimertinib, afatinib, gefitinib, erlotinib

## Abstract

First generation or second generation EGFR tyrosine kinase inhibitors are currently the standard of care for the first-line management of non-small cell lung cancer (NSCLC) patients with activating mutations within the kinase domain of the epidermal growth factor receptor gene (1, 2). Resistance to targeted therapy can develop after 9–11 months (3–8). Third generation inhibitors were developed to target the EGFR T790M clone, which is the most common dominant second site resistance mutation after first or second generation inhibitors. Osimertinib received full FDA approval for the second-line treatment of advanced NSCLC based on a phase III study comparing the compound to chemotherapy. Recent data demonstrates an important impact for osimertinib in the front-line space based on results comparing the compound to first-generation erlotinib or gefitinib therapy.

## The Story so Far

First and second generation EGFR tyrosine kinase inhibitors (EGFR TKIs) improve progression-free survival (PFS) from 5 to 11 months compared to chemotherapy in the front line (6, 9–11). Second generation inhibitors were created as an attempt to target the second site T790M mutation by irreversibly binding to the EGFR tyrosine kinase domain. Afatinib was developed as an irreversible EGFR/HER2 inhibitor designed to covalently bind to Cys 773 on the *EGFR* tyrosine kinase domain, and had improved inhibition of EGFR T790M in preclinical models (12, 13). However, in the LUX-LUNG 1 clinical trial, the response to afatinib after progression on erlotinib or gefitinib and chemotherapy was only 7% suggesting that use after progression on a first-generation TKI may be less efficacious than second-line chemotherapy alone. In the front line LUX-Lung 3 and LUX-Lung 6 trials, afatinib did not significantly improve overall survival (OS) versus chemotherapy (5, 14). Although a pre-specified subanalysis of each trial suggested a statistically significant improvement in OS in patients with the exon 19 del *EGFR* mutation (15), the LUX-LUNG 7 trial failed to identify a statistically significant superior OS with afatinib compared to gefitinib. Updated analysis of co-primary end points in LUX-LUNG 7 showed a superior time-to-treatment failure, PFS, overall response rate (ORR) for afatinib albeit with more diarrhea and rash toxicity compared to gefitinib which had higher transaminase elevation (16).

Osimertinib was developed to target the T790M clone and has irreversible binding affinity to the cysteine-797 residue at the ATP binding site of EGFR (17). Pre-clinically, the drug also inhibits cellular growth in EGFR exon 19del, L858R, and EGFRm(+)/T790M(+) mutant cell lines (18). The phase I AURA trial had an objective response rate in T790M positive NSCLC patients of 61%, and a median duration of PFS of 9.6 months (19). Two subsequent phase II trials confirmed these results in more than 400 patients with a PFS of approximately 11 months (20), and the FDA approved osimertinib under the breakthrough therapy designation.

The AURA3 phase III trial showed a greater than 70% ORR and 10.1 month PFS (HR 0.30 systemically and HR of 0.32 in the CNS) (21). The FDA granted fast-track approval based on these initial trial data in November 2015 and full approval in March 2017 for patients with metastatic *EGFR* T790M mutant positive NSCLC after progression on first or second generation anti-EGFR TKIs.

FLAURA (NCT02296125) is a phase III head-to-head trial that directly compared first-line osimertinib (80 mg daily) with standard first-line therapy with gefitinib or erlotinib in a total of 556 patients. The magnitude of improvement in the interim analysis was important (HR 0.46), with a superior PFS (18.9 versus 10.2 months) compared to standard of care (SOC) erlotinib and gefitinib (18.9 versus 10.2 months, 9 month PFS). A similar HR of 0.47 was seen in the CNS metastasis cohort and suggests encouraging CNS activity (22).

## Previous Head to Head Trials of EGFR TKIs

Several previous studies have compared EGFR inhibitors head-to-head, but have failed to drive a new Soc in this setting. Both the CTONG 0901 (3) trial which compared erlotinib with gefitinib in a Chinese patient population and the multi-national LUX-LUNG 7 which compared afatinib to gefitinib did not identify a clearly superior drug in terms of PFS, OS, or toxicity. In LUX-LUNG 7, the median OS with afatinib was 27.9 months compared to 24.5 months in patients who received gefitinib (HR 0.85; *P* = 0.19) with a higher ORR of 70% with afatinib versus 56% with geftinib (16, 23, 24). The higher RR in LUX-LUNG 7 with afatinib was met with more frequent treatment-related grade ≥3 AEs and included diarrhea (13.1 versus 1.3%), rash (9.4 versus 3.1%), and fatigue (5.6 versus 0%) (16). Dacomitinib, another irreversible pan-Her tyrosine kinase inhibitor, was compared head-to-head to gefitinib in the ARCHER 1050 trial with a greater median PFS (14.7 versus 9.2 months; HR 0.59, *p* < 0.0001). However, there were increased grade 3 toxicities with 66% of patients requiring dose reduction (25). In ARCHER 1050, there was a significant increase in dermatitis acneiform (13.7%), diarrhea (8.4%), increased ALT (8.5%), paronychia (7.5%) and stomatitis (3.5%) in the dacomitinib arm (26). A different third-generation inhibitor, ASP8273, was compared to erlotinib/gefitinib in the first-line SOLAR study, and the trial was discontinued based on results from an interim data analysis in the investigational arm (27).

## Clinical Outcomes with Osimertinib

Clinical efficacy with osimertinib has been documented in the first-line and second-line space. The treatment naïve cohort of the AURA I trial (NCT01802632) demonstrated a 19.3 month PFS for osimertinib and suggested a future role for the compound in treatment naïve patients with EGFR mutant lung cancer (28). Pre-clinical evidence suggests that T790M outgrowth may occur early or late, and that the suppression of resistance clones earlier in therapy may translate into improved PFS and time to treatment failure on the compound (18, 29, 30).

The interim data cutoff of June 2017 of the phase III FLAURA trial (NCT02296125) comparing first-line osimertinib with erlotinib/gefitinib demonstrated an improved PFS over Soc options in patients with and without CNS metastases. A response rate of 80% was noted with 3% of patients (7/279) achieving a complete response. The median PFS was longer with osimertinib than with SOC options (18.9 versus 10.2 months, HR 0.46, *p* < 0.001). The ORR was similar in the two groups (80% with osimertinib and 76% in the SOC group). The duration of response was 17.2 months with osimertinib versus 8.5 months with standard EGFR TKIs (22). OS data is currently awaiting full maturity.

## CNS Control

Brain recurrence is a major site of progression on EGFR TKIs given the challenging pharmacokinetics, drug efflux transporter mechanisms, and molecular weight (31). Afatinib and gefitinib have a CNS PFS of 7.2–7.4 months (19, 30). The promising early CNS data with osimertinib showed higher tissue concentration, higher blood brain barrier (BBB) penetration, and lower influence of efflux transporters when compared to gefitinib and afatinib (32). Evidence from the BLOOM study (NCT02228369) showed higher BBB penetration with CSF concentration supporting activity in patients with leptomeningeal disease (33). Second-line therapy in the AURA3 study showed a CNS ORR of 70% (21/30) with osimertinib and 31% with chemotherapy (34) with a median CNS PFS of 11.7 versus 5.6 months (HR 0.32; *p* = 0.004). The hazard ratio for systemic disease control and CNS control was similar in the FLAURA study supporting the preclinical data of high penetration across the BBB (35). The CNS ORR was 66 versus 43% in favor of osimertinib (*n* = 128, *p* = 0.01) with a shorter time to response of 6.2 versus 11.9 months. For the 22 evaluable patients receiving osimertinib, five complete responses were noted compared with none in the Soc arm (36).

## Toxicity of Osimertinib

Although there was no specific statistical comparison of safety data in grades 1 and 2 reported, osimertinib had lower rates of all grade and grade 3–4 adverse events compared to first generation EGFR TKIs (34 versus 45%) despite a longer median duration of exposure with osimertinib. A separation of the distribution of grade 1 and 2 toxicities would help to put into context the AE profile of gefitinib and erlotinib versus osimertinib. Osimertinib has less than 1% risk of grade 3 skin rash, paronychia, and stomatitis (17). Dose reductions in FLAURA were 5.4% and discontinuations were 13% which was favorable compared to other EGFR TKIs (22). LUX-LUNG 7 had 13% skin rash, 9% diarrhea, 2–4% paronychia and stomatitis with an overall dose reduction rate of 42.6% with afatinib (24). Importantly, while rare, an awareness of QTc prolongation and cardiomyopathy (with echocardiogram surveillance for patients with cardiac risk factors), keratitis, and interstitial lung disease are important considerations for patients on osimertinib therapy.

## Clinical Practice Recommendations

FLAURA has achieved an impressive triad of doubling of PFS, improved RR, and lower toxicity, and this serves as a compelling reason to consider osimertinib first-line therapy. This consideration also helps to address the nuanced issue of penetration of T790M testing in the real world setting which is disappointingly low at 16.8% overall. In certain regions testing at initial diagnosis for EGFR mutation remains quite low and may occur at a rate of 22.6% for stage IV adenocarcinoma patients (37). When an EGFR mutation is detected, some reports have documented that only 48.3% of stage IV patients will receive an EGFR TKI (37). The drop off in testing for mechanisms of resistance will be important in treatment selection for considering front-line use of osimertinib in EGFR mutated NSCLC patients.

Another concern that affects treatment selection decisions is the fact that almost 18% of the gefitinib and 36% afatinib patients did not receive further lines of treatment in the LUX-LUNG 7 trial (16). In the AURA 3 clinical trial, only 24% in the osimertinib group and 71% in the platinum-pemetrexed group received subsequent systemic treatment (21). Only 67% of advanced NSCLC patients overall receive a second-line therapy demonstrating the importance of patient drop off in clinical practice (16, 24).

## Limitations of Flaura

A balanced analysis of FLAURA does present some additional considerations. The patients in the Soc arm mainly received gefitinib, while erlotinib has been more prevalently utilized in the United States (38). It is not clear is how osimertinib would have compared to second generation irreversible inhibitors, and afatinib and dacomitinib have a non-significant numerical advantage in PFS compared to first generation TKIs erlotinib and gefitinib (39, 40).

Further investigation across large cohorts is needed to determine if the mechanisms of resistance to first-line osimertinib are unique. Based on second-line data, early progression on osimertinib may be more often associated with the development of alternate resistance mechanisms, such as MET upregulation, MEK activation, and small cell transformation among others. Patients with longer duration of response may stay addicted to EGFR with additional second site mutations noted, including C797S and others (41–43). The incidence of C797S resistance after first-line osimertinib is unknown at this time, and it remains to be determined if second-line first-generation inhibitors will be an adequate strategy against the C797S acquired resistance mutation. Evaluation of samples from the treatment naïve AURA patients and at progression revealed JAK2, PI3K, Her2 exon 20 insertions, and NOTCH mutations as acquired bypass mechanisms. Combined RB1 loss and p53 aberrations were identified in 3/19 patients by ctDNA (44). The possibility that this may select a pre-existing small cell clone is not yet known, and *EGFR* mutant SCLC transformed tumors frequently have p53, RB1, and PI3K aberrations (45–47). An MRI brain was not mandated at baseline in the FLAURA trial confounding the detection of asymptomatic cranial metastases on study (22). The optimal timing of osimertinib therapy will be further explored in the Phase II APPLE trial through the EORTC, in which first line osimertinib will be compared with osimertinib after gefitinib based on ctDNA progression.

## Role of Molecular Testing in Patient Selection

The limited dataset in the AURA 1 trial showed no cases of acquired T790M after progression on osimertinib in the first-line space (35). Currently, there is FDA approval for plasma Cobas testing for EGFR mutations when tissue is not available. Because of the rates of small cell lung cancer identified and a false negative rate in plasma, tissue testing will remain an important source for testing. Patients who had EGFR mutations identified by plasma ctDNA (359 patients) had a similar PFS to the full tissue positive set (15.2 months versus 9.7 months with SOC) (48).

The timing for surveillance of resistance clones may provide important information about disease biology (49). In the first assessment at 6 weeks, FLAURA showed an early separation of the PFS Kaplan–Meier curves which may indicate a lower frequency of early resistance to osimertinib (22). Monitoring for resistance mutations through plasma ctDNA will likely be a strategy forward for identifying resistance pathways.

## Overcoming C797S

Strategies to overcome resistance with C797 mutation are evolving. Chemotherapy is a standard option for those who progress on first-line osimertinib. In pre-clinical models, EGFR C797 mutations may respond to cetuximab and brigatinib, however, this remains to be tested in human clinical trials (50). When EGFR T790M and C797S are in the *cis* conformation (on the same allele), there are no active EGFR TKIs or combinations which have shown clinical responses in this setting to date (51, 52). Through plasma surveillance, it has been seen that C797S may exist in *trans* conformation (on different alleles) in approximately 8% of cases (52). There are reports of clinical efficacy with therapy combining first and third generation TKIs when T790M and C797S mutations are in the *trans* conformation. Wang et al. reported a short response with osimertinib and erlotinib targeting concomitant *EGFR* T790M and C797S in *trans*, and this was followed by a change in clonal dynamics in C797S from *trans* to *cis* (53). In another report in which T790M and C797S mutations were in *trans*, a ctDNA assay showed a rapid decline in the C797S mutation within 2 weeks of starting a gefitinib and osimertinib combination (54). These reports suggest the importance of making available the reporting of *cis* versus *trans* conformation for C797S after osimertinib therapy on sequencing reports to potentially guide therapies.

## Conclusion

Mounting clinical data supports that osimertinib will likely be a pivotal first-line treatment for EGFR mutant metastatic NSCLC. The FDA recently awarded breakthrough therapy designation to osimertinib in the first-line treatment of metastatic EGFR mutated NSCLC. The improvement in PFS, ORR, CNS efficacy, and toxicity demonstrate its important capacity as an important front-line option and have led the NCCN to recommend the compound in the first-line treatment of EGFR mutant patients. An important goal for EGFR mutant patients is to ensure early access to effective agents recognizing that not all patients will receive second-line therapy. Osimertinib is an attractive choice for CNS disease with early data on the prevention of CNS metastasis. The toxicity profile of the compound appears to be superior to other compounds in this space. Ongoing work to identify the mechanisms of secondary resistance to osimertinib can lead to rationale combinations of targeted therapy. The TATTON Phase Ib study evaluates combinations of osimertinib at increasing doses in combination with selumetinib (MEK inhibitor), AZD6094 (MET inhibitor) in T790M mutation-positive patients who have progressed *EGFR* tyrosine kinase inhibitor therapy harboring the T790M mutation. It is not entirely known if the mechanisms of resistance after second-line osimertinib will faithfully resemble all the mechanisms of resistance to first-line osimertinib, and this is an active area of ongoing research (NCT03122717).

## Author Contributions

Both authors were involved in the conception, design, and writing of the manuscript.

## Conflict of Interest Statement

HH received research funding from Pfizer and advisory board with Astra Zeneca and Abbvie, and speaker’s bureau with Bristol Myers Squibb, Merck, and Astrazeneca. AB is on advisory board of Pfizer and Astra Zeneca.

## Abbreviations

Mo, months; NA, not available; OS, overall survival; PFS, progression-free survival; ORR, overall response rate; Soc, standard of care; NSCLC, non-small cell lung cancer; HR, hazard ratio; cfDNA, cell-free DNA; ctDNA, circulating tumor DNA.
